# Cerebrospinal Fluid and Blood Neurofilament Light Chain Protein in Prion Disease and Other Rapidly Progressive Dementias: Current State of the Art

**DOI:** 10.3389/fnins.2021.648743

**Published:** 2021-03-12

**Authors:** Samir Abu-Rumeileh, Piero Parchi

**Affiliations:** ^1^Department of Neurology, Ulm University Hospital, Ulm, Germany; ^2^Istituto di Ricovero e Cura a Carattere Scientifico (IRCCS) Istituto delle Sciente Neurologiche di Bologna, Bologna, Italy; ^3^Department of Experimental Diagnostic and Specialty Medicine (DIMES), University of Bologna, Bologna, Italy

**Keywords:** Creutzfeldt-Jakob disease, prion disease, neurofilament light chain, Alzheimer’s disease, frontotemporal dementia, encephalitis, autoimmune, biofluid biomarkers

## Abstract

Rapidly progressive dementia (RPD) is an umbrella term referring to several conditions causing a rapid neurological deterioration associated with cognitive decline and short disease duration. They comprise Creutzfeldt–Jakob disease (CJD), the archetypal RPD, rapidly progressive variants of the most common neurodegenerative dementias (NDs), and potentially treatable conditions such as infectious or autoimmune encephalitis and cerebrovascular disease. Given the significant clinical and, sometimes, neuroradiological overlap between these different disorders, biofluid markers also contribute significantly to the differential diagnosis. Among them, the neurofilament light chain protein (NfL) has attracted growing attention in recent years as a biofluid marker of neurodegeneration due to its sensitivity to axonal damage and the reliability of its measurement in both cerebrospinal fluid (CSF) and blood. Here, we summarize current knowledge regarding biological and clinical implications of NfL evaluation in biofluids across RPDs, emphasizing CJD, and other prion diseases. In the latter, NfL demonstrated a good diagnostic and prognostic accuracy and a potential value as a marker of proximity to clinical onset in pre-symptomatic *PRNP* mutation carriers. Similarly, in Alzheimer’s disease and other NDs, higher NfL concentrations seem to predict a faster disease progression. While increasing evidence indicates a potential clinical value of NfL in monitoring cerebrovascular disease, the association between NfL and prediction of outcome and/or disease activity in autoimmune encephalitis and infectious diseases has only been investigated in few cohorts and deserves confirmatory studies. In the era of precision medicine and evolving therapeutic options, CSF and blood NfL might aid the diagnostic and prognostic assessment of RPDs and the stratification and management of patients according to disease progression in clinical trials.

## Introduction

Rapidly progressive dementia (RPD) is an umbrella term that comprehends several heterogenous diseases characterized by a rapidly progressive cognitive decline and a short disease duration, typically 1–2 years between onset and occurrence of dementia and/or of 2–3 years from onset to death (Cohen et al., 2015; Grau-Rivera et al., 2015; Geschwind and Murray, 2018; Rudge et al., 2018; Zerr and Hermann, 2018). Evidence from epidemiological studies indicates that RPD is not a rare condition. However, data concerning the actual prevalence are lacking. The most frequent causes of RPDs comprise prion disease and other neurodegenerative dementias (NDs), autoimmune and infectious encephalitis, vascular and metabolic encephalopathies, and malignancies (Geschwind and Murray, 2018; Zerr and Hermann, 2018).

Sporadic Creutzfeldt–Jakob’s disease (CJD) represents the prototype of RPDs and is often referred to as the “great mimicker” given its wide phenotypic heterogeneity (Baiardi et al., 2018; Geschwind and Murray, 2018). The extensive clinical overlaps between RPDs, the urgency and severity of the clinical scenario, and the existence of treatable forms make the availability of reliable diagnostic and prognostic biomarkers of primary importance. Brain magnetic resonance imaging (MRI), cerebrospinal fluid (CSF) surrogate markers such as proteins total (t)-tau and 14-3-3, and electroencephalographic (EEG) examination, support the clinical diagnosis of probable CJD according to current criteria (Zerr and Parchi, 2018; Rhoads et al., 2020). However, the overall sensitivity and specificity of these diagnostic investigations are not optimal. Indeed, a significant proportion of non-prion RPDs may show positivity in one or more of these tests, whereas, on the other hand, atypical disease subtypes with slower progression may escape recognition (Hamlin et al., 2012; Lattanzio et al., 2017; Rudge et al., 2018). In this regard, the prion real-time quacking-induced conversion (RT-QuIC) assay with its virtually full specificity for prion disease significantly contributed to the improvement of the diagnostic assessment of RPDs (Candelise et al., 2020). However, the assay is still not fully available and represents in many laboratories a second-step diagnostic assay to be applied when screening tests are suggestive for prion disease (Abu-Rumeileh et al., 2019a). Thus, the availability of sensitive and readily accessible analytes for large and rapid screening remains of critical importance. Besides, there is also urgent need for markers to use as surrogate end points in ongoing clinical trials showing positive associations with prognostic variables, such as overall survival, disease severity, and progression rate.

The assessment of neurofilament light chain protein (NfL) has attracted growing attention in recent years due to its sensitivity to neuronal damage and reliability of its measurement in both CSF and blood (Gaetani et al., 2019; Barro et al., 2020). NfL is a subunit of neurofilaments, which are cytoskeletal proteins mainly located in large myelinated axons where they play an important role in maintaining neuronal structure. After neuronal damage, NfL reaches the interstitial fluid, which communicates freely with the CSF and the blood (Gaetani et al., 2019). Several studies have explored the added values of this biomarker in the diagnostic and prognostic assessment of suspected CJD cases, with promising results. Moreover, given the high awareness of clinical neurologists for the treatable forms of RPD, particularly for autoimmune encephalitis (AE), attempts to evaluate the diagnostic and prognostic role of NfL have also been recently extended to this field.

In the present review, we summarize the current knowledge regarding the biological and clinical implications of CSF and blood NfL across the most prevalent etiologies of RPD.

## Prion Disease

Prion diseases or transmissible spongiform encephalopathies are rare, phenotypically heterogeneous, rapidly progressive neurodegenerative diseases (Parchi and Saverioni, 2012). The pathogenesis of prion diseases relies on the templated seeded conversion of the cellular prion protein (PrP^C^), encoded by the prion protein (*PRNP*) gene, into a pathological isoform with abnormal conformation (PrP^Sc^), which shows a tendency to aggregate and forms amyloid fibrils (Parchi and Saverioni, 2012; Puoti et al., 2012; Baiardi et al., 2019).

Sporadic CJD (sCJD) represents the most common form of human prion disease and accounts for about 85–90% of cases. The second most common variant (10–15% of cases) is genetic prion disease, which is related to pathogenic mutations in *PRNP* and encompasses three distinct clinical–pathological phenotypes, the genetic form of CJD (gCJD), fatal familial insomnia (FFI), and Gerstmann–Sträussler–Scheinker syndrome (GSS). Finally, a small proportion of cases (1–2%) is acquired (Baiardi et al., 2019). In turn, sCJD includes six major clinicopathological subtypes that are mainly determined by the genotype at the methionine (M)/valine (V) polymorphic codon 129 of the *PRNP* gene and the type (1 or 2) of PrP^Sc^ accumulating in the brain. The subtypes are named and classified according to these two main molecular variables into MM(V)1, MM2 cortical (MM2C), MM2 thalamic (MM2T), MV2 kuru (MV2K), VV1, and VV2 subtypes (Parchi et al., 1999, 2012; Baiardi et al., 2019).

### Diagnostic Value and Distribution Across Prion Disease Subtypes

Several studies (Table 1) evaluated CSF NfL in the prion disease spectrum. They showed significantly increased mean levels in most disease subtypes compared to non-neurodegenerative controls and other NDs (Steinacker et al., 2016; Kovacs et al., 2017; Abu-Rumeileh et al., 2018b, 2019a, 2020a; Zerr et al., 2018; Kanata et al., 2019; Vallabh et al., 2020). Interestingly, CSF NfL concentration varied significantly among sCJD subtypes and only partially correlated with t-tau levels (Abu-Rumeileh et al., 2018b; Zerr et al., 2018), suggesting that the two markers reflect distinct pathophysiological mechanisms. Both t-tau and NfL levels seem to be influenced by the neuronal degeneration occurring in a given period (i.e., speed of disease progression). Still, NfL raises more significantly than t-tau in the diseases with more widespread subcortical pathology (i.e., in deep nuclei, brainstem, cerebellum, and spinal cord), likely because of a more significant involvement of myelinated axons in white matter tracts in these regions compared to the cerebral cortex (Abu-Rumeileh et al., 2018b). Indeed, sCJD subtypes VV2 and MV2K showed significantly higher CSF NfL levels in comparison to the MM(V)1 group (Abu-Rumeileh et al., 2018b, 2020a; Zerr et al., 2018), which correlates with the more widespread and severe subcortical pathology and the higher amount of PrP^Sc^ accumulation and microglial activation in subcortical white matter in the former subtypes (Parchi et al., 2012; Baiardi et al., 2017; Franceschini et al., 2018). The divergent behavior of NfL and t-tau is especially evident in the MV2K subtype, which shows significantly lower concentrations of 14-3-3 and t-tau in CSF (Lattanzio et al., 2017) and a slower disease progression than the VV2. This might also explain the very high sensitivity of NfL for other slowly progressive prion disease subtypes showing low CSF values of t-tau and protein 14-3-3 [e.g., sCJD MM2C, gCJD E200K, GSS, FFI, and variable protease-sensitive prionopathy (VPSPr)] (Abu-Rumeileh et al., 2018b, 2019a; Zerr et al., 2018).

**TABLE 1:** Main findings of studies evaluating CSF and blood NfL in prion disease.

	Country of studied population	Examined biofluid	Assay	*N* according to prion disease form	*N* according to non-prion disease	AUC (cutoff)* sCJD vs. Controls	AUC (cutoff)* sCJD vs. non-prion	Other significant findings
Steinacker et al. (2016)	Germany	CSF, Blood	CSF: ELISA (IBL) Blood: Simoa	42 CJD (39 definite): 33 sCJD, 9 gCJD, 1 GSS (pre-symp)	20 dementia patients	CSF: 0.949 (>2,156 pg/ml) Blood: 0.959 (>44.7 pg/ml)		Elevated CSF NfL in one pre-symptomatic GSS case
Kovacs et al. (2017)	Austria	CSF, Blood	CSF: ELISA (Uman Dia.) Blood: Simoa	86 definite CJD: 65 sCJD, 21 gCJD	46 non-prion RPDs (all definite)	CSF: 0.979 Blood: 0.992	CSF: 0.451, vs. AD 0.768. Blood: 0.497, vs. AD 0.657.	No marked differences in blood and CSF NfL among sCJD subtypes
Abu-Rumeileh et al. (2018b)	Italy	CSF	CSF: ELISA (IBL)	141 prion (115 definite): 123 sCJD, 1 VPSPr 16 gCJD, 1 GSS	73 AD (37 rp), 35 DLB (11 rp), 44 FTLD (9 rp)	CSF: 1.00	0.926 vs. all. Atypical prion vs. rpNDs 0.839.	Significantly higher CSF levels in the sCJD subtypes VV2 and MV2K compared to the MM(V)1 group
Thompson et al. (2018)	United Kingdom	Blood	Blood: Simoa	45 sCJD (40 definite) 6 with serial samples	–	Blood: 1.00 (>44.7 pg/ml)	–	No correlation between blood NfL and rate of disease progression. Trend toward increased NfL in serial samples taken within 12 months from death
Zerr et al. (2018)	Germany, Poland, Spain, Portugal, Italy	CSF	CSF: ELISA (Uman Dia.)	314 prion (257 definite) Cohort 1: 112 sCJD Cohort 2: 20 sCJD 182 gCJD	Cohort 1: 11 MCI, 88 AD, 41 DLB/PDD, 36 VaD, 11 FTD. Cohort 2: 37 MCI, 20 AD, 12 DLB/PDD, 10 VaD, 30 FTD	CSF: 0.99 (>7,000 pg/ml)	0.90 overall (>10,500 pg/ml) >0.9 vs. AD, >0.9 vs. DLB/PDD, 0.83 vs. FTD; 0.76 vs. VaD	Prion disease: Significantly higher CSF levels in VV than in MM or MV codon 129 *PRNP* genotypes. Increase in NFL levels in consecutive LPs in cases with duration > 6 months. CSF NfL as a moderate prognostic marker.
Abu-Rumeileh et al. (2019a)	Italy	CSF	CSF: ELISA (IBL)	103 prion (103 definite): 80 sCJD, 1 VPSPr, 22 gCJD	109 non-prion RPDs	–	0.693 (>1,847 pg/ml)	CSF NfL most sensitive surrogate marker in virtually all prion diseases
Kanata et al. (2019)	Poland, Greece	CSF	CSF: ELISA (Uman Dia.)	Cohort 1: 43 CJD Cohort 2: 21 CJD	Cohort 1: 34. Cohort 2: 29	–	CSF: Cohort 1: 0.89 (>4,200 pg/ml), Cohort 2: 0.86 (>4,200 pg/ml)	
Staffaroni et al. (2019)	United States	CSF, Blood	CSF: ELISA (IBL) Blood: Simoa	188 CJD (147 definite)	–	–	–	CSF and blood NfL associated with survival (before adjusting for covariates)
Abu-Rumeileh et al. (2020a)	Italy	CSF, Blood	CSF: ELISA (IBL) Blood: Simoa	336 prion (254 definite): 275 sCJD, 1 VPSPr, 28 gCJD, 3 FFI, 3 GSS	106 non-prion-RPDs	–	CSF: 0.646 (>1,846 pg/ml). Blood: 0.616 (>87.9 pg/ml)	CSF and blood NfL associated with survival (even after adjusting for covariates). Blood NfL as strong prognostic factor in slowly progressive prion disease
Thompson et al. (2020)	United Kingdom	Blood	Blood: Simoa	231 sCJD, 14 iCJD, 17 vCJD 23 pre-symptomatic, 9 converting, 83 symptomatic *PRNP* mutation carriers	31 AD, 33 FTD, 24 non-prion RPDs	Blood: 1.00 (>25.87 pg/ml)	Blood: 0.912 vs. all (>60.65 pg/ml), 0.724 vs. non-prion RPDs (>60.65 pg/ml)	Blood NfL higher in sCJD compared to genetic prion disease and vCJD. Blood NfL correlates with severity of functional impairment but not with rate of disease progression. Higher blood NfL up to 2 years before onset but not earlier in pre-symptomatic *PRNP* mutation carriers
Vallabh et al. (2020)	United States, Australia	CSF, Blood	CSF: ELISA (Uman Dia., in-house) Blood: Simoa	27 pre-symptomatic *PRNP* mutation carriers 26 definite CJD: 24 sCJD, 2 gCJD	–	–	–	No difference in CSF and blood NfL between pre-symptomatic carriers and controls. No temporal increase in blood and CSF NfL in longitudinal assessments

*Cutoffs were reported in parentheses when available.

CSF NfL showed good diagnostic value across different studies on prion disease and full discrimination between CJD patients and controls (AUC 0.949–1.000) (Steinacker et al., 2016; Kovacs et al., 2017; Abu-Rumeileh et al., 2018b; Zerr et al., 2018). Interestingly, CSF NfL, either alone or in combination with other biomarkers, yielded a performance similar to t-tau in the distinction of prion disease from other NDs (AUC 0.926 vs. 0.939) and showed even a higher diagnostic value than t-tau in the specific comparisons between atypical prion disease and other rpNDs (AUC 0.839 vs. 0.722) (Abu-Rumeileh et al., 2018b). However, the biomarker accuracy for an early clinical diagnosis should be ideally assessed in a clinically or neuropathological based cohort of patients with heterogeneous RPD etiologies raising the clinical suspicion of prion disease, but only a few studies considered this approach (Kovacs et al., 2017; Abu-Rumeileh et al., 2019a, 2020a; Kanata et al., 2019). In this type of studies, CSF NfL diagnostic accuracy (AUC 0.451–0.890) was significantly lower than that of CSF t-tau (AUC 0.849–0.918) and/or protein 14-3-3 (AUC 0.711–0.908) (Kovacs et al., 2017; Abu-Rumeileh et al., 2018b, 2020a; Kanata et al., 2019). Indeed, increased levels of NfL are detected in most vascular, and neuroinflammatory diseases, and even in neurodegenerative disorders commonly associated with a slower progressive course than RPDs. However, NfL levels overlapping significantly with those seen in CJD mainly characterize frontotemporal dementia, amyotrophic lateral sclerosis, and atypical parkinsonism (Gaetani et al., 2019). Therefore, given its low specificity, CSF NfL has a limited role as an isolated test in the differential diagnosis of RPDs (Abu-Rumeileh et al., 2019a, 2020a; Kanata et al., 2019). Nevertheless, when specific tests such as RT-QuIC are also adopted in series, CSF NfL might be useful as a first step screening assay for suspected CJD cases as an alternative to t-tau or 14-3-3.

More recently, blood NfL levels were also found significantly higher in patients with prion disease belonging to all CJD forms and subtypes than both controls and other NDs (Table 1; Steinacker et al., 2016; Kovacs et al., 2017; Thompson et al., 2018, 2020; Abu-Rumeileh et al., 2020a). However, in contrast to the CSF analyte, blood NfL levels did not significantly differ among the most prevalent sCJD subtypes (Kovacs et al., 2017; Thompson et al., 2018; Abu-Rumeileh et al., 2020a). Interestingly, sCJD VV2, typically characterized by the highest CSF NfL and t-tau values among sCJD subtypes (Lattanzio et al., 2017; Abu-Rumeileh et al., 2018b, 2020a; Zerr et al., 2018), showed similar blood NfL and reduced blood tau levels compared with the MM(V)1 type (Kovacs et al., 2017; Abu-Rumeileh et al., 2020a). These data suggest that the different regional lesion profiles between the two CJD subtypes (Parchi et al., 1999; Baiardi et al., 2017), particularly the early cortical neuronal damage featuring MM(V)1 but not VV2, might be responsible for a higher spill over of these molecules in the blood compared to other brain regions (Abu-Rumeileh et al., 2020a). An overview of the distribution of CSF and blood NfL and a comparison with other classic and new biofluid markers across distinct prion disease forms and subtypes are provided in Table 2.

**TABLE 2:** Distribution of CSF and blood markers across prion disease subtypes.

	Sporadic CJD				Genetic prion disease			
Matrix–Marker (reference)	MM(V)1	VV2	MV2K	MM2C	gCJD E200K	gCJD V210I	FFI	GSS
**Neuronal/axonal damage**								
CSF NfL (Abu-Rumeileh et al., 2018b*, 2019a*; Kovacs et al., 2017; Zerr et al., 2018)	↑↑	↑↑↑	↑↑(↑)	↑↑	↑↑	↑↑	↑(↑)	↑↑
Blood NfL (Kovacs et al., 2017; Abu-Rumeileh et al., 2020a)	↑↑	↑↑	↑↑	↑	↑↑	↑↑	↑	↑
CSF t-tau (Lattanzio et al., 2017*; Abu-Rumeileh et al., 2018b*, 2019a*, 2020a*)	↑↑(↑)	↑↑↑	↑(↑)	↑(↑)	↑↑	↑↑(↑)	(↑)	↑(↑)
Blood tau (Kovacs et al., 2017; Abu-Rumeileh et al., 2020a)	↑↑↑	↑(↑)	↑(↑)	↑	↑↑(↑)	↑↑(↑)	(↑)	=
CSF 14-3-3 (Lattanzio et al., 2017*; Abu-Rumeileh et al., 2019a*, 2020a*)	↑↑(↑)	↑↑↑	↑	↑	↑↑	↑↑(↑)	(↑)	NA
**Neuroinflammation**								
CSF YKL-40 (Llorens et al., 2017b; Abu-Rumeileh et al., 2019b*)	↑↑	↑↑↑	↑↑	↑(↑)	↑↑	↑↑	↑(↑)	↑↑
CSF GFAP (Abu-Rumeileh et al., 2019b*)	↑	↑↑	↑	↑	↑	(↑)	(↑)	↑
CSF CHIT1 (Abu-Rumeileh et al., 2019b*)	↑↑	↑↑↑	↑↑↑	↑(↑)	↑(↑)	↑	↑↑	↑↑(↑)
**Synaptic damage**								
CSF α-synuclein (Llorens et al., 2017a, 2018)	↑↑(↑)**	↑↑(↑)**	NA	NA	↑↑	↑↑	=	=
CSF neurogranin (Blennow et al., 2019)	↑↑	↑	NA	NA	NA	NA	NA	NA
**Other mechanisms**								
CSF t-PrP (Villar-Piqué et al., 2019)	↓↓	↓↓	↓↓	NA	↓↓	↓	↓↓	=
Blood t-PrP (Llorens et al., 2020b)	↑↑	↑↑	↑	↑↑	↑↑	↑↑(↑)	↑↑	NA
CSF p-tau (Lattanzio et al., 2017*, Abu-Rumeileh et al., 2018b*)	(↑)	↑↑	↑	=	(↑)	(↑)	NA	NA
CSF ubiquitin (Abu-Rumeileh et al., 2020b)	↑↑	↑↑↑	↑	↑	↑	↑↑	=	↑

*We considered only recent studies (2017–2020) and prion disease subtypes, in which at least one of the reported fluid biomarkers was analyzed in at least 10 patients.****↑****indicates increased values in comparison to controls. The number of arrows expresses the degree of increase* (***↑****: mild*, ***↑↑****: moderate*, ***↑↑↑***: severe), the bracket indicates intermediate levels between two degrees. ↓ decreased values in comparison to controls with the same semiquantitative scale. =, values within the normal range. NA: data not available. *CSF t-tau, NfL, 14-3-3 and p-tau data from some patients were included in multiple studies. **Only data on MM and VV genotypes are available. However, >95% of VV cases are VV2 and >95% of MM cases are MM1 (or MM1+2C).

Regarding the diagnostic value of blood NfL, only three studies to date evaluated the marker in clinically and/or neuropathological heterogeneous cohorts of RPDs (Kovacs et al., 2017; Abu-Rumeileh et al., 2020a; Thompson et al., 2020). They obtained similar results, namely, the lower value of blood NfL compared to blood tau in the discrimination between prion (or sCJD) and non-prion RPDs (NfL: AUC 0.497–0.724 vs. tau: AUC 0.722–0.837) (Kovacs et al., 2017; Abu-Rumeileh et al., 2020a; Thompson et al., 2020). Notably, the performance of classic CSF markers such as CSF t-tau and 14-3-3 appeared significantly superior to both plasma tau and NfL, raising the question of the real utility of blood surrogate markers in the diagnostic assessment of RPDs (Abu-Rumeileh et al., 2020a).

In conclusion, the very high negative predictive value of NfL for prion disease, due to its high sensitivity for neural damage, may justify its use as an early screening marker. Indeed, low or normal CSF and/or blood levels of NfL in a patient with RPD might exclude with very high certainty the diagnosis of prion disease and induce clinicians to consider other etiologies and to introduce an *ex adiuvantibus* therapy. In contrast, a significant increase of its levels in at least one biofluid should prompt a second more specific test such as the RT-QuIC.

### Prognostic Value

Different methods have been adopted to assess the rate of clinical progression in prion disease. The MRC Prion Disease Rating Scale, a validated scale of functional impairment, has been used to model linear slopes of functional decline as a measure of the rate of clinical progression (Thompson et al., 2013). Despite the lack of a significant association between NfL values and the rate of disease progression (MRC slope), Thompson et al. (2018, 2020) found a slight correlation between blood NfL values and the degree of functional impairment, along with a tendency toward blood NfL levels raising over time in serial samples of symptomatic patients with increasingly functional impairment. Moreover, they found elevated blood NfL concentrations in all disease stages, with no overlapping values with controls in every sample tested longitudinally, suggesting a potential role of the marker as an outcome measure in clinical trials (Thompson et al., 2020). Similarly, in another study, CSF and blood NfL levels significantly correlated with another scale (i.e., Barthel Index) of functional impairment (Staffaroni et al., 2019).

To date, only three studies explored the association between NfL in CSF and/or blood and survival in prion disease (Zerr et al., 2018; Staffaroni et al., 2019; Abu-Rumeileh et al., 2020a). Zerr and colleagues originally reported that CSF NfL performs only moderately as a prognostic marker (Zerr et al., 2018). However, two studies later showed that both CSF and blood NfL were significantly associated with survival in prion disease/CJD (Staffaroni et al., 2019; Abu-Rumeileh et al., 2020a). However, only Abu-rumeileh et al. stratified the analysis according to the disease subtype, a strong independent factor in prion disease. The collected data showed that while blood and/or CSF t-tau best predicted survival in sCJD MM(V)1 and VV2 subgroups, blood NfL (but not CSF NfL) was significantly associated with survival in the slowly progressive prion diseases. Although a test for *in vivo* PrP^Sc^ typing is currently unavailable, codon 129 genotyping allows partial patient stratification since early disease onset. In this regard, multivariate analyses adjusted for age and codon 129 genotype showed that CSF and blood NfL, CSF tau, and CSF 14-3-3 were still predictive of survival (Staffaroni et al., 2019; Abu-Rumeileh et al., 2020a; Llorens et al., 2020a). Therefore, current evidence strongly recommends a stratification according to disease subtype or at least codon 129 genotype (in the clinical setting) to select outcomes and endpoints in future clinical trials (Abu-Rumeileh et al., 2020a).

### Biomarker Values Throughout the Disease Course of Genetic Prion Disease

Any future treatment aimed to prevent prion disease cannot prescind from the identification and longitudinal evaluation of pre-symptomatic *PRNP* mutation carriers. The significant heterogeneity of the age at onset in the prion disease spectrum combined with the rarity of the disease strongly limits the use of clinical onset as an outcome in preventive clinical trials. In this regard, identifying a biomarker of proximity to disease onset might be critical to overcome the issue (Minikel et al., 2019, 2020; Vallabh et al., 2020).

To date, only two studies explored neurofilament dynamics in cross-sectional and/or longitudinal samples from pre-symptomatic and symptomatic *PRNP* mutation carriers. One reported no difference in CSF and/or blood NfL levels between pre-symptomatic *PRNP* mutation carriers and healthy controls and no temporal increase in longitudinal blood and CSF samples in pre-symptomatic cases (Vallabh et al., 2020). Similarly, in the study of Thompson et al. (2020), NfL concentrations did not differ between pre-symptomatic *PRNP* mutation carriers with samples collected more than 2 years before symptom onset and those of controls. However, the marker increased progressively in the longitudinal evaluation from 2 years from onset to the symptomatic phase. In summary, the available data on this aspect are, to some extent, controversial. They potentially reflect a common selection bias, namely, the inclusion of subjects with several different *PRNP* mutations and heterogeneous clinical progression rates.

Nevertheless, recent evidence indicates that blood NfL may perform well as a surrogate endpoint of response to antisense oligonucleotides (ASOs) therapy in a prion disease mouse model (Minikel et al., 2020), where a significant reduction of the biomarker level was documented after drug administration. These findings resembled those obtained in the II phase trial for ASOs in SOD1 ALS (Miller et al., 2020) and open new possible horizons for implementing blood NfL as a reliable and accessible marker of disease monitoring in clinical trials for neurodegenerative diseases.

## Other Neurodegenerative Dementias

NDs represent the most common etiology in non-prion RPD patients’ cohorts and a frequent source of CJD misdiagnosis (Geschwind and Murray, 2018; Zerr and Hermann, 2018). In this regard, rapidly progressive variants of AD (rpAD), dementia with Lewy bodies (DLB), and frontotemporal dementia (FTD) may represent distinct subtypes of these disorders characterized by rapid cognitive decline, short disease duration (1–3 years), and subtle distinctive neuropathological and/or biochemical features (Cohen et al., 2015; Drummond et al., 2017; Lee et al., 2017; Abu-Rumeileh et al., 2018a; Geut et al., 2019; Zerr and Hermann, 2018).

The differential diagnosis between CJD and rp-NDs is still challenging, given the partial overlap in clinical features and CSF levels of the surrogate protein markers 14-3-3 and total-tau (Abu-Rumeileh et al., 2018b; Geschwind and Murray, 2018). For this purpose, other CSF biomarkers, including NfL (Abu-Rumeileh et al., 2018b), were tested, especially in the comparison between prion disease and rpAD. However, the evaluation of NfL levels in slowly and rp-ND forms failed to reveal a significant difference between the two groups, apart from FTD (Abu-Rumeileh et al., 2018b).

Another crucial open issue concerns the *a priori* identification of subjects with faster cognitive decline in AD and FTD cohorts to improve patient stratification for future clinical trials. In this regard, few studies showed an association between high CSF NfL levels and rapid cognitive decline in AD’s mild cognitive impairment stage (Zetterberg et al., 2016; Pillai et al., 2020). Similarly, in the FTD spectrum, higher basal CSF and/or blood NfL values have been related to faster disease progression (in some clinical syndromes) (Ljubenkov et al., 2018; Rojas et al., 2018) and shorter survival (Meeter et al., 2018; van der Ende et al., 2019; Benussi et al., 2020; Cajanus et al., 2020), suggesting the utility of an early NfL assessment in the prediction of future disease aggressiveness.

## Autoimmune Encephalitis and Other Potentially Treatable Causes of RPD

AE represents an increasingly recognized common cause of RPD (Geschwind and Murray, 2018).

Apart from the relevant diagnostic issue of the exclusion of prion disease (Abu-Rumeileh et al., 2019a), CSF and blood NfL have been explored in AE as easily accessible tools for disease monitoring, prognosis, and response to therapy. Although data are scanty and inconsistent, some findings deserve to be mentioned. In analogy with other RPDs, patients with AE typically showed higher CSF and/or blood NfL values than controls or reference intervals (Constantinescu et al., 2016, 2017; Li et al., 2019; Mariotto et al., 2019). However, concentrations in the normal range have also been reported (Körtvelyessy et al., 2018).

Interestingly, patients on active disease/progression, those on recovery and/or after disease treatment, and those with a stable clinical picture seem to show increased, decreased, or steady levels, respectively (Constantinescu et al., 2016; Li et al., 2019; Mariotto et al., 2019). Given the good correlation between CSF NfL and the disability grade (Constantinescu et al., 2016; Li et al., 2019), these data suggest a role for NfL as a marker of disease activity in AE.

CSF NfL concentration also predicted long-term outcomes in patients with AE (Constantinescu et al., 2016, 2017; Macher et al., 2020). However, most studies showed no associations between NfL values, CSF parameters, and MRI variables (Constantinescu et al., 2016; Mariotto et al., 2019; Macher et al., 2020), reflecting a possible discrepancy between clinical severity and radiological or biochemical features in these conditions (Mariotto et al., 2019).

On a significant issue, all the studies mentioned above merged patients with several subtypes of AE, raising concerns about a possible selection bias that may have influenced the results discussed above.

In RPDs secondary to infectious etiologies, CSF NfL has been reported to predict long-term neurocognitive status in herpes simplex encephalitis (Westman et al., 2020). In contrast, the measurement of NfL concentrations in patients with varicella-zoster encephalitis showed discordant results between studies (Eckerström et al., 2020; Tyrberg et al., 2020). Moreover, despite an inconsistent correlation between NfL levels and degree of cognitive impairment, NfL concentrations have been reported to increase 2 years before the appearance of cognitive decline in patients with HIV–dementia complex, suggesting a possible prognostic role for the marker (Gisslén et al., 2007; McLaurin et al., 2019).

Interestingly, NfL data have also been recently obtained in cerebrovascular disease forms that are occasionally associated with a rapid course, such as small vessel disease. Here, blood NfL concentrations have been associated with disability and neurological symptoms, including future cognitive impairment (Gattringer et al., 2017; Duering et al., 2018; Peters et al., 2020). Moreover, the marker correlated with disease severity, disease progression, and survival in CADASIL patients (Gravesteijn et al., 2018).

## Discussion and Concluding Remarks

Here, we have provided an overview of currently available CSF and blood NfL data on RPDs. Evidence suggests the potential value of NfL as a first-step diagnostic test in the assessment of RPD patients to quickly screen for the presence of ongoing neuronal damage before performing more specific and/or expensive investigations such as RT-QuIC and/or MRI. Moreover, NfL measurement in biofluids might be useful to track the disease trajectory in prion disease and other neurodegenerative dementias. In this regard, higher levels of CSF and/or blood protein seemed to predict a faster disease progression, higher grade of functional impairment, and/or shorter survival, which are all relevant variables for the stratification and management of patients in ongoing clinical trials. Furthermore, preliminary evidence suggests that blood NfL may have a role as a marker of proximity to clinical onset and, possibly, as the surrogate endpoint of response to ASOs in pre-symptomatic *PRNP* mutation carriers, which are currently the best candidates for new therapeutic options. While in cerebrovascular disease, the evidence of an association between NfL, disease severity, and/or outcome is growing, the role of the marker in AE and infectious encephalitis is not well-clarified, given that current data sets include small cohorts of heterogeneous etiologies and/or insufficient data. In the era of precision medicine, the imminent application of biofluid markers in the routine flow chart may significantly improve the diagnostic and prognostic assessment of patients with RPD.

## Author Contributions

SA-R and PP assembled all the data and wrote the manuscript. Both authors contributed to the article and approved the submitted version.

## Conflict of Interest

The reviewer PH declared a past co-authorship with one of the authors PP to the handling editor. The author declares that the research was conducted in the absence of any commercial or financial relationships that could be construed as a potential conflict of interest.

## References

[B1] Abu-Rumeileh S. Baiardi S. Ladogana A. Zenesini C. Bartoletti-Stella A. Poleggi A. (2020a). Comparison between plasma and cerebrospinal fluid biomarkers for the early diagnosis and association with survival in prion disease. *J. Neurol. Neurosurg. Psychiatry* 91 1181–1188. 10.1136/jnnp-2020-323826 32928934

[B2] Abu-Rumeileh S. Baiardi S. Polischi B. Mammana A. Franceschini A. Green A. (2019a). Diagnostic value of surrogate CSF biomarkers for Creutzfeldt-Jakob disease in the era of RT-QuIC. *J. Neurol.* 266 3136–3143. 10.1007/s00415-019-09537-0 31541342

[B3] Abu-Rumeileh S. Capellari S. Parchi P. (2018a). Rapidly Progressive Alzheimer’s Disease: Contributions to Clinical-Pathological Definition and Diagnosis. *J. Alzheimers Dis.* 63 887–897. 10.3233/JAD-171181 29710713

[B4] Abu-Rumeileh S. Capellari S. Stanzani-Maserati M. Polischi B. Martinelli P. Caroppo P. (2018b). The CSF neurofilament light signature in rapidly progressive neurodegenerative dementias. *Alzheimers Res. Ther.* 10:3. 10.1186/s13195-017-0331-1 29368621 PMC5784714

[B5] Abu-Rumeileh S. Oeckl P. Baiardi S. Halbgebauer S. Steinacker P. Capellari S. (2020b). CSF Ubiquitin Levels Are Higher in Alzheimer’s Disease than in Frontotemporal Dementia and Reflect the Molecular Subtype in Prion Disease. *Biomolecules* 10:497. 10.3390/biom10040497 32218217 PMC7226617

[B6] Abu-Rumeileh S. Steinacker P. Polischi B. Mammana A. Bartoletti-Stella A. Oeckl P. (2019b). CSF biomarkers of neuroinflammation in distinct forms and subtypes of neurodegenerative dementia. *Alzheimers Res. Ther.* 12:2. 10.1186/s13195-019-0562-4 31892365 PMC6937795

[B7] Baiardi S. Capellari S. Bartoletti Stella A. Parchi P. (2018). Unusual Clinical Presentations Challenging the Early Clinical Diagnosis of Creutzfeldt-Jakob Disease. *J. Alzheimers Dis.* 64 1051–1065. 10.3233/JAD-180123 30010123

[B8] Baiardi S. Magherini A. Capellari S. Redaelli V. Ladogana A. Rossi M. (2017). Towards an early clinical diagnosis of sporadic CJD VV2 (ataxic type). *J. Neurol. Neurosurg. Psychiatry* 88 764–772. 10.1136/jnnp-2017-315942 28668775

[B9] Baiardi S. Rossi M. Capellari S. Parchi P. (2019). Recent advances in the histo-molecular pathology of human prion disease. *Brain Pathol.* 29 278–300. 10.1111/bpa.12695 30588685 PMC8028685

[B10] Barro C. Chitnis T. Weiner H. L. (2020). Blood neurofilament light: a critical review of its application to neurologic disease. *Ann. Clin. Transl. Neurol.* 7 2508–2523. 10.1002/acn3.51234 33146954 PMC7732243

[B11] Benussi A. Karikari T. K. Ashton N. Gazzina S. Premi E. Benussi L. (2020). Diagnostic and prognostic value of serum NfL and p-Tau181 in frontotemporal lobar degeneration. *J. Neurol. Neurosurg. Psychiatry* 91 960–967. 10.1136/jnnp-2020-323487 32611664

[B12] Blennow K. Diaz-Lucena D. Zetterberg H. Villar-Pique A. Karch A. Vidal E. (2019). CSF neurogranin as a neuronal damage marker in CJD: a comparative study with AD. *J. Neurol. Neurosurg. Psychiatry* 90 846–853. 10.1136/jnnp-2018-320155 31097472

[B13] Cajanus A. Katisko K. Kontkanen A. Jääskeläinen O. Hartikainen P. Haapasalo A. (2020). Serum neurofilament light chain in FTLD: association with C9orf72, clinical phenotype, and prognosis. *Ann. Clin. Transl. Neurol.* 7 903–910. 10.1002/acn3.51041 32441885 PMC7318100

[B14] Candelise N. Baiardi S. Franceschini A. Rossi M. Parchi P. (2020). Towards an improved early diagnosis of neurodegenerative diseases: the emerging role of in vitro conversion assays for protein amyloids. *Acta Neuropathol. Commun.* 8:117. 10.1186/s40478-020-00990-x 32711575 PMC7382043

[B15] Cohen M. L. Kim C. Haldiman T. ElHag M. Mehndiratta P. Pichet T. (2015). Rapidly progressive Alzheimer’s disease features distinct structures of amyloid-. *Brain* 138 1009–1022. 25688081 10.1093/brain/awv006 PMC5014074

[B16] Constantinescu R. Krýsl D. Andrén K. Asztély F. Bergquist F. Zetterberg H. (2017). Cerebrospinal fluid markers of neuronal and glial cell damage in patients with autoimmune neurologic syndromes with and without underlying malignancies. *J. Neuroimmunol.* 306 25–30. 10.1016/j.jneuroim.2017.02.018 28385184

[B17] Constantinescu R. Krýsl D. Bergquist F. Andrén K. Malmeström C. Asztély F. (2016). Cerebrospinal fluid markers of neuronal and glial cell damage to monitor disease activity and predict long-term outcome in patients with autoimmune encephalitis. *Eur. J. Neurol.* 23 796–806. 10.1111/ene.12942 26822123

[B18] Drummond E. Nayak S. Faustin A. Pires G. Hickman R. A. Askenazi M. (2017). Proteomic differences in amyloid plaques in rapidly progressive and sporadic Alzheimer’s disease. *Acta Neuropathol.* 133 933–954. 10.1007/s00401-017-1691-0 28258398 PMC5503748

[B19] Duering M. Konieczny M. J. Tiedt S. Baykara E. Tuladhar A. M. Leijsen E. V. (2018). Serum Neurofilament Light Chain Levels Are Related to Small Vessel Disease Burden. *J. Stroke* 20 228–238. 10.5853/jos.2017.02565 29886723 PMC6007291

[B20] Eckerström M. Nilsson S. Zetterberg H. Blennow K. Grahn A. (2020). Cognitive impairment without altered levels of cerebrospinal fluid biomarkers in patients with encephalitis caused by varicella-zoster virus: a pilot study. *Sci. Rep.* 10:22400. 10.1038/s41598-020-79800-2 33372192 PMC7769988

[B21] Franceschini A. Strammiello R. Capellari S. Giese A. Parchi P. (2018). Regional pattern of microgliosis in sporadic Creutzfeldt-Jakob disease in relation to phenotypic variants and disease progression. *Neuropathol. Appl. Neurobiol.* 44 574–589. 10.1111/nan.12461 29345730

[B22] Gaetani L. Blennow K. Calabresi P. Di Filippo M. Parnetti L. Zetterberg H. (2019). Neurofilament light chain as a biomarker in neurological disorders. *J. Neurol. Neurosurg. Psychiatry* 90 870–881. 10.1136/jnnp-2018-320106 30967444

[B23] Gattringer T. Pinter D. Enzinger C. Seifert-Held T. Kneihsl M. Fandler S. (2017). Serum neurofilament light is sensitive to active cerebral small vessel disease. *Neurology* 89 2108–2114. 10.1212/WNL.0000000000004645 29046363 PMC5711505

[B24] Geschwind M. D. Murray K. (2018). Differential diagnosis with other rapid progressive dementias in human prion diseases. *Handb. Clin. Neurol.* 153 371–397. 10.1016/B978-0-444-63945-5.00020-9 29887146

[B25] Geut H. Vergouw L. J. M. Galis Y. Ingrassia A. de Jong F. J. Quadri M. (2019). Neuropathological and genetic characteristics of a post-mortem series of cases with dementia with Lewy bodies clinically suspected of Creutzfeldt-Jakob’s disease. *Parkinsonism Relat. Disord.* 63 162–168. 10.1016/j.parkreldis.2019.02.011 30777654

[B26] Gisslén M. Hagberg L. Brew B. J. Cinque P. Price R. W. Rosengren L. (2007). Elevated cerebrospinal fluid neurofilament light protein concentrations predict the development of AIDS dementia complex. *J. Infect. Dis.* 195 1774–1778. 10.1086/518043 17492593

[B27] Grau-Rivera O. Gelpi E. Nos C. Gaig C. Ferrer I. Saiz A. (2015). Clinicopathological Correlations and Concomitant Pathologies in Rapidly Progressive Dementia: A Brain Bank Series. *Neurodegener. Dis.* 15 350–360. 10.1159/000439251 26523804

[B28] Gravesteijn G. Rutten J. W. Verberk I. M. W. Böhringer S. Liem M. K. van der Grond J. (2018). Serum Neurofilament light correlates with CADASIL disease severity and survival. *Ann. Clin. Transl. Neurol.* 6 46–56. 10.1002/acn3.678 30656183 PMC6331956

[B29] Hamlin C. Puoti G. Berri S. Sting E. Harris C. Cohen M. (2012). A comparison of tau and 14-3-3 protein in the diagnosis of Creutzfeldt-Jakob disease. *Neurology* 79 547–552. 10.1212/WNL.0b013e318263565f 22843257 PMC4098812

[B30] Kanata E. Golanska E. Villar-Piqué A. Karsanidou A. Dafou D. Xanthopoulos K. (2019). Cerebrospinal fluid neurofilament light in suspected sporadic Creutzfeldt-Jakob disease. *J. Clin. Neurosci.* 60 124–127. 10.1016/j.jocn.2018.09.031 30309804

[B31] Körtvelyessy P. Prüss H. Thurner L. Maetzler W. Vittore-Welliong D. Schultze-Amberger J. (2018). Biomarkers of Neurodegeneration in Autoimmune-Mediated Encephalitis. *Front. Neurol.* 9:668. 10.3389/fneur.2018.00668 30283395 PMC6156245

[B32] Kovacs G. G. Andreasson U. Liman V. Regelsberger G. Lutz M. I. Danics K. (2017). Plasma and cerebrospinal fluid tau and neurofilament concentrations in rapidly progressive neurological syndromes: a neuropathology-based cohort. *Eur. J. Neurol.* 24 1326–e77. 10.1111/ene.13389 28816001

[B33] Lattanzio F. Abu-Rumeileh S. Franceschini A. Kai H. Amore G. Poggiolini I. (2017). Prion-specific and surrogate CSF biomarkers in Creutzfeldt-Jakob disease: diagnostic accuracy in relation to molecular subtypes and analysis of neuropathological correlates of p-tau and Aβ42 levels. *Acta Neuropathol.* 133 559–578. 10.1007/s00401-017-1683-0 28205010 PMC5348556

[B34] Lee E. B. Porta S. Michael Baer G. Xu Y. Suh E. Kwong L. K. (2017). Expansion of the classification of FTLD-TDP: distinct pathology associated with rapidly progressive frontotemporal degeneration. *Acta Neuropathol.* 134 65–78. 10.1007/s00401-017-1679-9 28130640 PMC5521959

[B35] Li J. Gu Y. An H. Zhou Z. Zheng D. Wang Z. (2019). Cerebrospinal fluid light and heavy neurofilament level increased in anti-N-methyl-d-aspartate receptor encephalitis. *Brain Behav.* 9:e01354. 10.1002/brb3.1354 31313506 PMC6710226

[B36] Ljubenkov P. A. Staffaroni A. M. Rojas J. C. Allen I. E. Wang P. Heuer H. (2018). Cerebrospinal fluid biomarkers predict frontotemporal dementia trajectory. *Ann. Clin. Transl. Neurol.* 5 1250–1263. 10.1002/acn3.643 30349860 PMC6186942

[B37] Llorens F. Kruse N. Karch A. Schmitz M. Zafar S. Gotzmann N. (2018). Validation of α-Synuclein as a CSF Biomarker for Sporadic Creutzfeldt-Jakob Disease. *Mol. Neurobiol.* 55 2249–2257. 10.1007/s12035-017-0479-5 28321768 PMC5840235

[B38] Llorens F. Kruse N. Schmitz M. Gotzmann N. Golanska E. Thüne K. (2017a). Evaluation of α-synuclein as a novel cerebrospinal fluid biomarker in different forms of prion diseases. *Alzheimers Dement.* 13 710–719. 10.1016/j.jalz.2016.09.013 27870938

[B39] Llorens F. Rübsamen N. Hermann P. Schmitz M. Villar-Piqué A. Goebel S. (2020a). A prognostic model for overall survival in sporadic Creutzfeldt-Jakob disease. *Alzheimers Dement.* 16 1438–1447. 10.1002/alz.12133 32614136

[B40] Llorens F. Thüne K. Tahir W. Kanata E. Diaz-Lucena D. Xanthopoulos K. (2017b). YKL-40 in the brain and cerebrospinal fluid of neurodegenerative dementias. *Mol. Neurodegener.* 12:83. 10.1186/s13024-017-0226-4 29126445 PMC5681777

[B41] Llorens F. Villar-Piqué A. Schmitz M. Diaz-Lucena D. Wohlhage M. Hermann P. (2020b). Plasma total prion protein as a potential biomarker for neurodegenerative dementia: diagnostic accuracy in the spectrum of prion diseases. *Neuropathol. Appl. Neurobiol.* 46 240–254. 10.1111/nan.12573 31216593

[B42] Macher S. Zrzavy T. Höftberger R. Altmann P. Pataraia E. Zimprich F. (2020). Longitudinal measurement of CSF neurofilament light in anti-NMDAR encephalitis. *Eur. J. Neurol.* 2020:14631. 10.1111/ene.14631 33145945 PMC7984371

[B43] Mariotto S. Gajofatto A. Zuliani L. Zoccarato M. Gastaldi M. Franciotta D. (2019). Serum and CSF neurofilament light chain levels in antibody-mediated encephalitis. *J. Neurol.* 266 1643–1648. 10.1007/s00415-019-09306-z 30944980

[B44] McLaurin K. A. Booze R. M. Mactutus C. F. (2019). Diagnostic and prognostic biomarkers for HAND. *J. Neurovirol.* 25 686–701. 10.1007/s13365-018-0705-6 30607890 PMC6610810

[B45] Meeter L. H. H. Vijverberg E. G. Del Campo M. Rozemuller A. J. M. Donker Kaat L. de Jong F. J. (2018). Clinical value of neurofilament and phospho-tau/tau ratio in the frontotemporal dementia spectrum. *Neurology* 90 1231–1239e. 10.1212/WNL.0000000000005261 29514947 PMC5890612

[B46] Miller T. Cudkowicz M. Shaw P. J. Andersen P. M. Atassi N. Bucelli R. C. (2020). Phase 1-2 Trial of Antisense Oligonucleotide Tofersen for SOD1 ALS. *N. Engl. J. Med.* 383 109–119. 10.1056/NEJMoa2003715 32640130

[B47] Minikel E. V. Vallabh S. M. Orseth M. C. Brandel J. P. Haïk S. Laplanche J. L. (2019). Age at onset in genetic prion disease and the design of preventive clinical trials. *Neurology* 93 125–134e. 10.1212/WNL.0000000000007745 31171647 PMC6656649

[B48] Minikel E. V. Zhao H. T. Le J. O’Moore J. Pitstick R. Graffam S. (2020). Prion protein lowering is a disease-modifying therapy across prion disease stages, strains and endpoints. *Nucleic Acids Res.* 48:gkaa616. 10.1093/nar/gkaa616 32776089 PMC7641729

[B49] Parchi P. Saverioni D. (2012). Molecular pathology, classification, and diagnosis of sporadic human prion disease variants. *Folia Neuropathol.* 50 20–45. 22505361

[B50] Parchi P. de Boni L. Saverioni D. Cohen M. L. Ferrer I. Gambetti P. (2012). Consensus classification of human prion disease histotypes allows reliable identification of molecular subtypes: an inter-rater study among surveillance centres in Europe and USA. *Acta Neuropathol.* 124 517–529. 10.1007/s00401-012-1002-8 22744790 PMC3725314

[B51] Parchi P. Giese A. Capellari S. Brown P. Schulz-Schaeffer W. Windl O. (1999). Classification of sporadic Creutzfeldt-Jakob disease based on molecular and phenotypic analysis of 300 subjects. *Ann. Neurol.* 46 224–233. 10443888

[B52] Peters N. van Leijsen E. Tuladhar A. M. Barro C. Konieczny M. J. Ewers M. (2020). Serum Neurofilament Light Chain Is Associated with Incident Lacunes in Progressive Cerebral Small Vessel Disease. *J. Stroke* 22 369–376. 10.5853/jos.2019.02845 33053952 PMC7568975

[B53] Pillai J. A. Bena J. Bebek G. Bekris L. M. Bonner-Jackson A. Kou L. (2020). Inflammatory pathway analytes predicting rapid cognitive decline in MCI stage of Alzheimer’s disease. *Ann. Clin. Transl. Neurol.* 7 1225–1239. 10.1002/acn3.51109 32634865 PMC7359114

[B54] Puoti G. Bizzi A. Forloni G. Safar J. G. Tagliavini F. Gambetti P. (2012). Sporadic human prion diseases: molecular insights and diagnosis. *Lancet Neurol.* 11 618–628. 10.1016/S1474-4422(12)70063-7 22710755

[B55] Rhoads D. D. Wrona A. Foutz A. Blevins J. Glisic K. Person M. (2020). Diagnosis of prion diseases by RT-QuIC results in improved surveillance. *Neurology* 95 1017–1026e. 10.1212/WNL.0000000000010086 32571851

[B56] Rojas J. C. Bang J. Lobach I. V. Tsai R. M. Rabinovici G. D. Miller B. L. (2018). CSF neurofilament light chain and phosphorylated tau 181 predict disease progression in PSP. *Neurology* 90 273–281e. 10.1212/WNL.0000000000004859 29282336 PMC5798651

[B57] Rudge P. Hyare H. Green A. Collinge J. Mead S. (2018). Imaging and CSF analyses effectively distinguish CJD from its mimics. *J. Neurol. Neurosurg. Psychiatry* 89 461–466. 10.1136/jnnp-2017-316853 29142140 PMC5909756

[B58] Staffaroni A. M. Kramer A. O. Casey M. Kang H. Rojas J. C. Orrú C. D. (2019). Association of Blood and Cerebrospinal Fluid Tau Level and Other Biomarkers With Survival Time in Sporadic Creutzfeldt-Jakob Disease. *JAMA Neurol.* 76 969–977. 10.1001/jamaneurol.2019.1071 31058916 PMC6503575

[B59] Steinacker P. Blennow K. Halbgebauer S. Shi S. Ruf V. Oeckl P. (2016). Neurofilaments in blood and CSF for diagnosis and prediction of onset in Creutzfeldt-Jakob disease. *Sci. Rep.* 6:38737. 10.1038/srep38737 27929120 PMC5144074

[B60] Thompson A. G. B. Luk C. Heslegrave A. J. Zetterberg H. Mead S. H. Collinge J. (2018). Neurofilament light chain and tau concentrations are markedly increased in the serum of patients with sporadic Creutzfeldt-Jakob disease, and tau correlates with rate of disease progression. *J. Neurol. Neurosurg. Psychiatry* 89 955–961. 10.1136/jnnp-2017-317793 29487167 PMC6109239

[B61] Thompson A. G. Anastasiadis P. Druyeh R. Whitworth I. Nayak A. Nihat A. (2020). Evaluation of plasma tau and neurofilament light chain biomarkers in a 12-year clinical cohort of human prion diseases. *medRxiv* 2020:20157594. 10.1038/s41380-021-01045-w PMC8758487 33674752

[B62] Thompson A. G. Lowe J. Fox Z. Lukic A. Porter M. C. Ford L. (2013). The Medical Research Council prion disease rating scale: a new outcome measure for prion disease therapeutic trials developed and validated using systematic observational studies. *Brain* 136 1116–1127. 10.1093/brain/awt048 23550114

[B63] Tyrberg T. Nilsson S. Blennow K. Zetterberg H. Grahn A. (2020). Serum and cerebrospinal fluid neurofilament light chain in patients with central nervous system infections caused by varicella-zoster virus. *J. Neurovirol.* 26 719–726. 10.1007/s13365-020-00889-2 32816287 PMC7532135

[B64] Vallabh S. M. Minikel E. V. Williams V. J. Carlyle B. C. McManus A. J. Wennick C. D. (2020). Cerebrospinal fluid and plasma biomarkers in individuals at risk for genetic prion disease. *BMC Med.* 18:140. 10.1186/s12916-020-01608-8 32552681 PMC7302371

[B65] van der Ende E. L. Meeter L. H. Poos J. M. Panman J. L. Jiskoot L. C. Dopper E. G. P. (2019). Serum neurofilament light chain in genetic frontotemporal dementia: a longitudinal, multicentre cohort study. *Lancet Neurol.* 18 1103–1111. 10.1016/S1474-4422(19)30354-0 31701893

[B66] Villar-Piqué A. Schmitz M. Lachmann I. Karch A. Calero O. Stehmann C. (2019). Cerebrospinal Fluid Total Prion Protein in the Spectrum of Prion Diseases. *Mol. Neurobiol.* 56 2811–2821. 10.1007/s12035-018-1251-1 30062673

[B67] Westman G. Aurelius E. Ahlm C. Blennow K. Eriksson K. Lind L. (2020). Cerebrospinal fluid biomarkers of brain injury, inflammation and synaptic autoimmunity predict long-term neurocognitive outcome in herpes simplex encephalitis. *Clin. Microbiol. Infect.* 2020:31. 10.1016/j.cmi.2020.09.031 32979577

[B68] Zerr I. Hermann P. (2018). Diagnostic challenges in rapidly progressive dementia. *Expert Rev. Neurother.* 18 761–772. 10.1080/14737175.2018.1519397 30173564

[B69] Zerr I. Parchi P. (2018). Sporadic Creutzfeldt-Jakob disease. *Handb. Clin. Neurol.* 153 155–174. 10.1016/B978-0-444-63945-5.00009-X 29887134

[B70] Zerr I. Schmitz M. Karch A. Villar-Piqué A. Kanata E. Golanska E. (2018). Cerebrospinal fluid neurofilament light levels in neurodegenerative dementia: Evaluation of diagnostic accuracy in the differential diagnosis of prion diseases. *Alzheimers Dement.* 14 751–763. 10.1016/j.jalz.2017.12.008 29391125

[B71] Zetterberg H. Skillbäck T. Mattsson N. Trojanowski J. Q. Portelius E. Shaw L. M. (2016). Association of Cerebrospinal Fluid Neurofilament Light Concentration With Alzheimer Disease Progression. *JAMA Neurol.* 73 60–67. 10.1001/jamaneurol.2015.3037 26524180 PMC5624219

